# Novel method for rapid in-situ hybridization of HER2 using non-contact alternating-current electric-field mixing

**DOI:** 10.1038/srep30034

**Published:** 2016-07-22

**Authors:** Yoshitaro Saito, Kazuhiro Imai, Ryuta Nakamura, Hiroshi Nanjo, Kaori Terata, Hayato Konno, Yoichi Akagami, Yoshihiro Minamiya

**Affiliations:** 1 Department of Thoracic Surgery, Akita University Graduate School of Medicine, 1-1-1 Hondo, Akita, Akita 010-8543, Japan; 2 Akita Industrial Technology Center, Akita, Japan; 3 Division of Clinical Pathology, Akita University Graduate School of Medicine, 1-1-1 Hondo, Akita, Akita 010-8543, Japan.

## Abstract

Human epidermal growth factor receptor 2 (HER2)-targeted agents are an effective approach to treating HER2-positive breast cancer patients. However, the lack of survival benefit in HER2-negative patients as well as the toxic effects and high cost of the drugs highlight the need for accurate and prompt assessment of HER2 status. Our aim was to evaluate the clinical utility of a novel rapid dual *in-situ* hybridization (RISH) method developed to facilitate hybridization. The method takes advantage of the non-contact mixing effect of an alternating current (AC) electric field. One hundred sixty-three specimens were used from patients diagnosed with primary breast cancers identified immunohistochemically as HER2 0/1(+), (2+) or (3+). The specimens were all tested using conventional dual *in-situ* hybridization (DISH), DISH with an automated slide stainer, and RISH. With RISH the HER2 test was completed within 6 h, as compared to 20–22 h needed for the standard protocol. Although RISH produced results more promptly using smaller amounts of labeled antibody, the staining and accuracy of HER2 status evaluation with RISH was equal to or greater than with DISH. These results suggest RISH could be used as a clinical tool to promptly determine HER2 status.

Breast cancer subtypes defined by their estrogen receptor (ER), progesterone receptor (PR), or human epidermal growth factor receptor 2 (HER2) status likely arise via different carcinogenic mechanisms. HER2 (also referred to as ErbB2/neu) is a proto-oncogene located on the long arm of chromosome 17 and encodes a transmembrane receptor of the epidermal growth factor receptor (EGFR) family^1^. Amplification of HER2 occurs in approximately 20% of breast cancers and is associated with shortened survival^2^,^3^. Trastuzumab and Pertuzumab, two humanized monoclonal antibodies that target HER2, and Lapatinib, a reversible dual inhibitor of the HER1 and HER2 tyrosine kinase, are routinely used to treat patients with breast cancers that overexpress HER2^2^,^3^,^4^,^5^,^6^,^7^. These HER2-targeted agents are an effective therapeutic approach for patients with HER2-positive metastatic breast cancer, but are ineffective in HER2-negative patients.

Immunohistochemistry (IHC) to assess overexpression of HER2 protein and *in situ* hybridization (ISH) to assess amplification of HER2 gene are currently the preferred techniques for evaluating HER2 status. IHC has little advantage over ISH with respect to sensitivity or the specificity of the antibodies used. However, whereas IHC can be performed with nearly every pathology and is comparatively inexpensive, ISH is time-consuming and requires the use of expensive probes and a special fluorescence microscopy facility, and has to be evaluated in the dark if using fluorescence ISH (FISH). Importantly, the majority of IHC 2+ cases, which account for 10–15% of all breast cancer patients, are responsive to HER2-targeted therapy, followed by IHC 3+ cases^1^,^8^,^9^. But IHC 2+ cases are often difficult to interpret because approximately 21% (range: 8–44.4%) exhibit HER2 amplification^8^. Evaluation of “non-informative” HER2 IHC 2+ tests therefore requires further analysis using a secondary gene amplification test such as FISH, dual-color ISH (DISH), chromogenic *in situ* hybridization (CISH), silver *in situ* hybridization (SISH) or multiplex ligation-dependent probe amplification (MLPA). The accurate and prompt evaluation of HER2 status has resulted in detection of a significantly higher number of cases with HER2 amplification, especially IHC 2+ cases, which has in turn led to more patients being identified as appropriate for targeted treatment.

We recently developed a rapid-IHC method that makes use of an alternating current (AC) electric field to facilitate the antigen-antibody reaction, and reported its usefulness for detection of lung cancer metastasis, central nervous system tumors, and mammalian eggs^10^,^11^,^12^. The device reduces the time required for IHC as well as the amount of antibody required for these analyses. With this device, we apply a high-voltage, low-frequency AC electric field to the sections. The resultant coulomb force stirs the antibody solution on the sections. This rapid-IHC method enables rapid detection of target cells in frozen sections and can provide a surgeon with an intraoperative diagnosis within about 30 min^11^. Although the rapid-IHC method still has limitations, as it has not yet been tested in other organs or with other detection methods, we anticipate that this technique will be applicable in multiple ways, for example in the hybridization step of DISH.

The aim of the present study was to evaluate the clinical utility, reliability and sensitivity of a novel rapid dual *in-situ* hybridization (RISH) method that promotes hybridization by taking advantage of the non-contact mixing effect in microdroplets subjected to an AC electric field.

## Results

**Figure 1:**
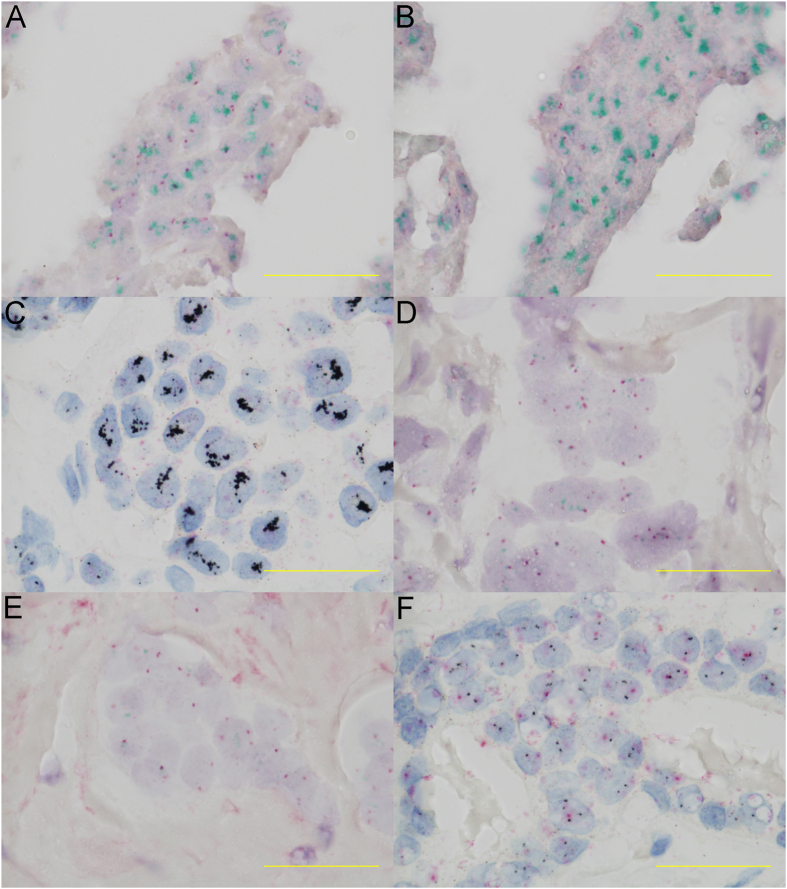
Detection of Breast Cancer Human Epidermal Growth Factor Receptor 2 (HER2) using conventional Dual-color *in Situ* Hybridization (DISH), rapid DISH (RISH), and DISH using an automated slide stainer. (**A**) Positive according the ASCO/CAP scoring criteria demonstrated using DISH. (**B**) Positive demonstrated using RISH. (**C**)Positive demonstrated using an automated slide stainer. (**D**) Negative demonstrated using DISH. (**E**) Negative demonstrated using RISH. (**F**) Negative demonstrated using an automated slide stainer. Red signals represent chromosome 17 centromere; green signals represent the HER2/neu gene locus at 17q12. Scale bar represents 50 μm.

HER2 scoring using IHC was performed with all specimens from enrolled breast cancer patients. The HER2 status of the 163 specimens that had HER2 0/(1+) or (2+), which were categorized as equivocal, or (3+) was evaluated using both DISH and RISH. Using IHC, 36 specimens were scored 0, 41 were scored (1+), 59 were scored (2+) and 27 were scored (3+). DISH using the standard Zytovision protocol or the new RISH protocol and DISH using an automated slide stainer all performed equally well (Fig. 1 and Supplementary Figure 1). On the other hand, RISH enabled detection of HER2 gene amplification within 6 h, which is much less time than was required for conventional DISH (Table 1). HER2 and/or chromosome enumeration probe (CEP) 17 signals were observed in 159 specimens (97.5%) when the Zytovison DISH protocol was used for staining, 163 specimens (100%) when RISH was used, and 162 specimens (99.4%) when the DISH automated slide stainer was used. Table 2 shows each HER2 status defined as the HER2/CEP17 ratio and average HER2 copy number for the 163 specimens. When the Zytovison DISH protocol was used, 133 specimens (81.6%) were evaluated as negative, and 26 (15.95%) were evaluated as amplified. Four specimens could not be evaluated because of poor HER2 and/or CEP17 staining. Twenty-five (92.6%) of the 27 specimens scored (3+) using IHC were evaluated as amplified, but 2 specimens were evaluated as negative. When RISH was used, 133 specimens (81.6%) were evaluated as negative, 30 (18.4%) were evaluated as amplified, and all 27 specimens scored (3+) using IHC were evaluated as amplified. When the automated slide stainer was used for DISH, 131 specimens (80.4%) were evaluated as negative, and 30 (18.4%) were evaluated as amplified. Two specimens were not testable. And all 27 specimens scored (3+) using IHC were evaluated as amplified.

We found 95.7% agreement between DISH and RISH based on the amplification status (Cohen kappa coefficient= 0.859, 95% confidence interval (CI): 0.760–0.958). Moreover, the agreement was 98.8% between the automated DISH slide stainer and RISH (Cohen kappa coefficient= 0.960, 95% CI 0.906–1.000).

The 13 patients categorized as HER2-positive using RISH received the HER2-targeted agents. The response rate to those agents was 84.6%, with 2 of 13 HER2 RISH-positive patients achieving a complete response, 9 achieving a partial response, and 2 maintaining stable disease.

## Discussion

In the present study, we demonstrated that RISH performed with a high-voltage, low-frequency AC electric field and the device can be used to detect HER2 amplification within 6 h. In addition, although RISH provides a result more promptly than other methods, specimens subjected to DISH or RISH were stained equally well, and the accuracy of the evaluation of HER2 gene amplification using RISH was equal to or greater than that obtained with conventional DISHs.

The high level of agreement between IHC and ISH means that neither is superior to the other as a predictor of the response to HER2-targeted treatment^1^,^8^,^9^,^13^, though testing gene amplification using ISH is more reliable. However, the ISH protocol has several well-known disadvantages: it is time-consuming, technically demanding, costly, and may delay the final determination of HER2 status. The standard ISH protocol requires approximately 22 h to complete, as hybridization usually takes 16–18 h. By contrast, RISH enabled the hybridization step to be finished in less than 3 h.

The lack of survival benefit of Trastuzumab, Pertuzumab and Lapatinib in HER2-negative tumors, as well as their toxic side effects and high cost, highlight the need for accurate assessment of HER2 status in all patients with invasive breast cancer^14^. Furthermore, according to the ASCO/CAP guidelines^1^,^15^, approximately 20% of results currently obtained using HER2 tests (IHC, ISH) are inaccurate. One of the main causes is likely the heterogeneous expression of HER2 observed among different patients with breast cancer, as well as between matched samples from primary tumors and their metastases^16^,^17^,^18^. Genetic heterogeneity is an additional phenomenon that can frequently result in discrepant or equivocal FISH, DISH, and IHC results across different tumor sections and metastatic lesions, affecting the accurate assessment of HER2 status and the determination of appropriate therapy^17^. Relatively common alterations that may affect the diagnosis of HER2 amplification include heterogeneity of HER2 amplification and deletion of sub-chromosomal regions containing the HER2 and/or CEP17 gene^16^. Although true polysomy 17 is very rare in breast carcinomas^19^,^20^, loss of the CEP17 region without loss of the HER2 gene locus could lead to a false diagnosis of HER2 gene amplification. However these disadvantages related to gene heterogeneity are common among all high-throughput molecular profiling techniques, including IHC, ISH, qPCR and microarray.

There is no consensus as to which protein- or gene-based HER2 testing technique is the best, though a second line ISH is regarded as necessary for all IHC 2+ patients^1^,^2^,^8^,^9^. Each technique, including ISH, has its own advantages and disadvantages, and there is always some discrepancy between the results obtained using different techniques. The response to HER2-targeted agents is the best end point, but not all methods have been tested individually, let alone in comparative studies^1^. Standardization, proper quality control assessment, laboratory accreditation, processing automation (including the hybridized step in RISH in a broad sense) and interpretation methods will all play important roles in HER2 testing.

It is also important to consider the effect of electroporation when using the RISH technique. Pores are created when an electric current is applied to the cell membrane, which raises the possibility of false positives and over-diagnosis. In our earlier report, we showed that the electric current does not flow through the inside of the microdroplet during AC electric field mixing^12^. This is because the stirred microdroplet is separated from the electrode by both glass and air, which act as insulators.

Intraoperative frozen sections are often required for reasons such as the need for rapid histological diagnosis and the need to intraoperatively assess surgical margin positivity or sentinel lymph node metastasis. Unfortunately, many ISH probes have been optimized for use with formalin-fixed, paraffin-embedded tissue, and fixation in 10% neutrally buffered formalin takes 24 h. Consequently, the new RISH method may be very useful for rapid intraoperative analysis as well as for use with very small pre-surgical samples, if RISH can be used with frozen samples. Further investigation will be needed to more precisely define the utility of RISH with frozen sections.

In summary, we have shown that rapid dual *in-situ* hybridization (RISH) can be used as a clinical tool for prompt determination of HER2 status in breast cancer samples. Using RISH with a high-voltage, low-frequency AC electric field, HER2 tests can be completed within 6 h, as compared to 20–22 h needed for conventional DISH. However, further investigation using other ISH methods will be needed to confirm the usefulness of this method.

## Methods

### Patients and Specimens

All experimental protocols were approved by the institutional review board at Akita University Hospital (Permit number: 1408), and written informed consent was obtained from all patients. The methods in this study were carried out in accordance with the approved guidelines. Patients who had undergone needle biopsy and/or radical surgery were enrolled in the study between April 2012 and March 2014 and were analyzed retrospectively. The patients’ characteristics are listed in Table 3. One hundred sixty-three specimens from patients diagnosed with primary breast cancer were identified as HER2 0/(1+), (2+) or (3+) using IHC and were used in this study. Of those, 116 specimens were biopsy samples and 47 were surgical samples. Using standard histological techniques, the specimens were fixed in 10–20% formalin, embedded in paraffin, cut at 3–4 μm, transferred to slides, and stained using hematoxylin/eosin and IHC. In addition, the specimens were subjected to conventional DISH using the routine Zytovision protocol, DISH using an automated slide stainer, and our RISH protocol.

### Standard Immunohistochemistry

IHC was performed using a BenchMark XT Staining Platform (Ventana Medical Systems, Tucson, AZ) with an automatic staining protocol. Standard IHC for HER2 was performed to make a decision whether to treat with Trastuzumab. An anti-HER2/neu (4B5) rabbit monoclonal antibody (Roche Diagnostics, Penzberg, Germany) was used as the primary antibody with the appropriate dilution. Reacted antibodies were visualized enzymatically using brown diaminobenzidene (DAB) as a substrate.

### HER2 Scoring system

The intensity and pattern of membrane immunoreactivity were evaluated by two independent pathologists in accordance with the new recommendations of the American Society of Clinical Oncology (ASCO)/College of American Pathologists (CAP)^15^. If the initial HER2 test of a core needle biopsy specimen from a primary breast cancer was negative, a new HER2 test was ordered for the excised specimen. We first tested for HER2 using a validated IHC assay. If the HER2 IHC test reported a score greater than HER2 (2+) as equivocal, we ordered and additional ISH using same specimen.

### Dual color *in situ* hybridization (DISH)

DISH was performed using a DNA-specific dual-color probes ZytoDot2C SPEC HER2/CEN 17 Probe Kit (ZytoVision, Bremerhaven, Germany). Sections were deparaffinized and incubated first for 5 min in 3% H_2_O_2_ to block endogenous peroxidase, and then for 15 min in a prewarmed pretreatment EDTA solution at 95–100 °C in a water bath. After washing the sections in distilled water, 100 μl of pepsin solution was applied to the slides, which were then incubated for 5 min at room temperature in a humidity chamber. Thereafter, the sections were washed in distilled water and dehydrated through an ethanol series. Ten microliters of the ZytoDot 2C SPEC HER2/CEN 17 Probe (P) was applied to each slide, after which the sections were covered with a coverslip and sealed with Parafilm^®^M (SIGMA, USA). The samples were then denatured at 80 °C for 5 min, transferred to a humidity chamber and hybridized overnight (16–18 h) in a hybridization oven at 37 °C. After hybridization, immunodetection was performed according to the manufacturer’s instructions, and the sections were counterstained with hematoxylin and mounted. HER2 expression was assessed by examination under a microscope.

### DISH using an automated slide stainer

INFORM HER2 Dual ISH DNA Probe Assays were performed on a BenchMark XT Staining Platform (Ventana Medical Systems, Tucson, AZ). The run protocol was established so that the entire assay procedure, consisting of baking, deparaffinization, pretreatment, hybridization, stringency wash, signal detection and counterstaining, was completed as a one-step fully automated assay. Each assay was finished within 13.5 h on this system.

### New rapid dual color *in situ* hybridization (RISH)

**Figure 2:**
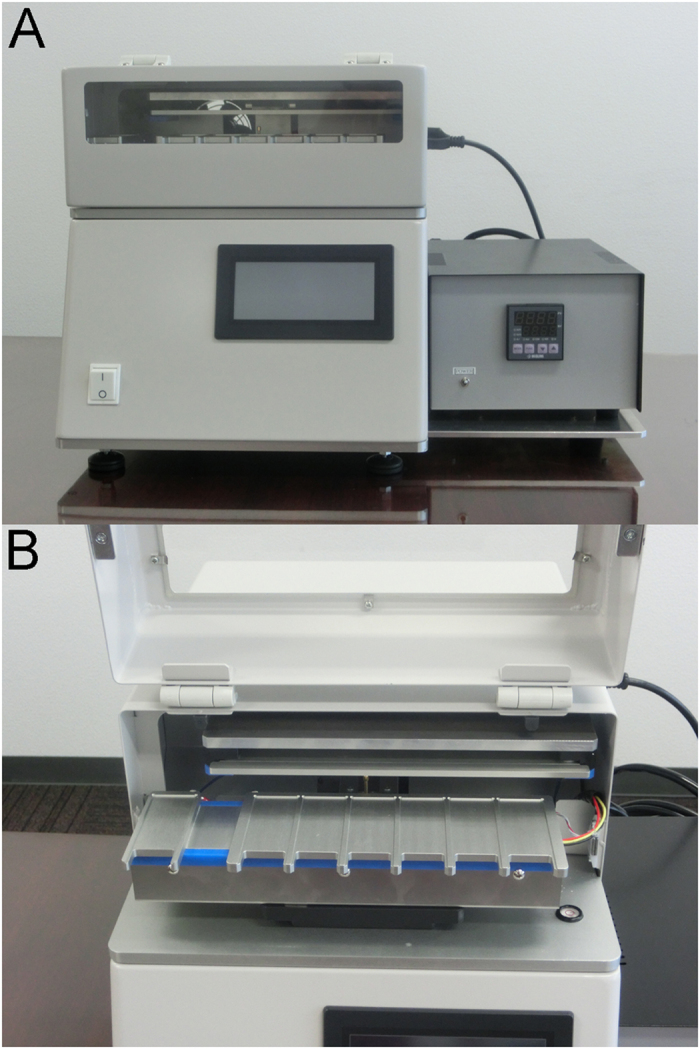
Device used to apply a high-voltage, low-frequency AC electric field. The resultant coulomb force stirs the DNA probe solution on the sections. (**A**) The device for rapid Dual-color *in Situ* Hybridization. (**B**) The microscope slide between the electrodes

**Figure 3:**
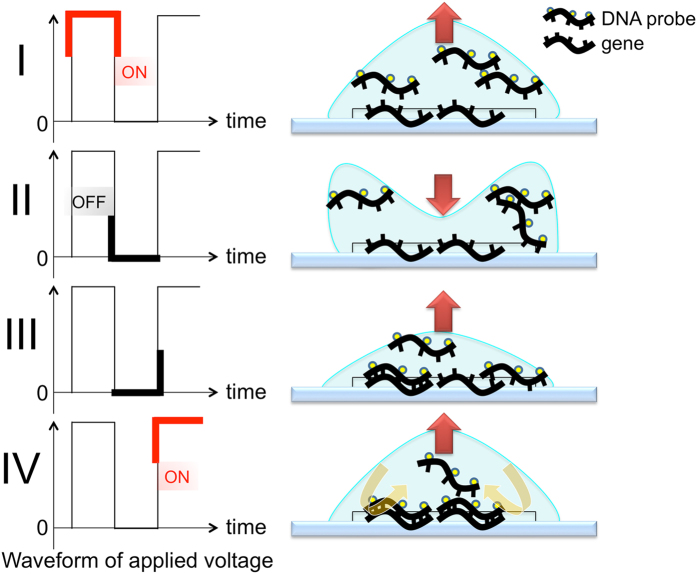
Schema of the changes in the microdroplet as the voltage is switched on and off. The DNA probes are mixed within the microdroplet as the voltage is switched on and off in a time series (I→II→III→IV). The resultant coulomb force stirs the probe solution on the sections, and the opportunity for contact between the probe and gene is increased because as the voltage is turned on and off at regular intervals, the microdroplet’s shape is transformed.

We used the prototype for RISH, which has been already reported in the previous paper^10^, after mounting with the temperature control unit (Fig. 2). The theory behind and technique for AC electric field mixing was described in detail previously^10^,^11^,^12^. After mounting an insoluble label cover (SLS/E-bar rabel II, Roche Diagnostics, Penzberg, Germany, with a 1-cm diameter hole in the center) on each microscope slide, 10 μl of ZytoDot 2C SPEC HER2/CEN 17 Probe (P) were applied evenly. Thereafter, 40 μl of HAIKORU-K140N (KANEDA, Japan), which is a very low viscosity liquid paraffin (4.0–5.5 mm^2^/s), were added as an oil cover to prevent probe evaporation. The slide was placed between the electrodes and a high-voltage (4.5 KV, offset 2.4 KV), low-frequency (15 Hz) AC current was applied (Fig. 3 and Supplementary Video 1). There was a distance of 3.8–4.0 mm between the slide and electrode plates, and the current was applied for 3 h at 37 °C. Anti-DIG/DNP mix (30 μl) and HRP/AP polymer mix (30 μl) were applied for 20 min under the same voltage and frequency conditions after the slide was covered with liquid paraffin.

Table 1 summarizes each procedure and the time required for ISH. The HER2 amplification based on the dual probe HER2/CEP 17 ratio with an average HER2 copy number and signals/cell was evaluated according the ASCO/CAP scoring criteria^15^ in both the DISH and RISH protocol.

### Statistical analysis

Statistical analysis was performed using JMP IN 10.0.2 software (SAS Institute, Cary, NC, USA). Cohen’s kappa-coefficient was used to assess agreement of 4 × 2-contingency tables between protocols. A kappa-value < 0.4 indicates fair to poor agreement, 0.4–0.8 indicates moderate to good agreement, and >0.8 indicates excellent agreement.

## Additional Information

**How to cite this article**: Saito, Y. *et al.* Novel method for rapid in-situ hybridization of HER2 using non-contact alternating-current electric-field mixing. *Sci. Rep.* **6**, 30034; doi: 10.1038/srep30034 (2016).

## Supplementary Material

Supplementary Figure 1

Supplementary Video 1

## Figures and Tables

**Table 1:** Procedural details for DISH, RISH and DISH by automated slides stainer.

Protocol	DISH	RISH	DISH using ASS
Dewaxing, activation, dehydration	1 h	1 h	1 h
Denaturation and hybridization	16–18 h (37 °C)	3 hours (37 °C), AC	8 h (40 °C)
Anti-DIG/DNP	30 min	20 min, AC	150 min
AP/HRP	30 min	20 min, AC	
AP chromogenic reaction	30 min	15 min	
HRP chromogenic reaction	30 min	15 min	
Washing slides and other step	1 h	1 h	2 h
Total time	20–22 h	6 h	13.5 h

DIG, digoxigenin; DNP, dintrophenyl; AP, alkaline phosphatase: HRP, horseradish peroxidase; AC, alternating current electric field; ASS, Automated slide stainer.

**Table 2:** HER2 results based on DISH, RISH and DISH using an automated slide stainer.

HER2 IHC	Case	DISH		RISH		DISH using ASS	
0	36	Negative	36	Negative	36	Negative	36
		Equivocal	0	Equivocal	0	Equivocal	0
		Positive	0	Positive	0	Positive	0
		Not evaluable	0	Not evaluable	0	Not evaluable	0
1+	41	Negative	40	Negative	41	Negative	41
		Equivocal	0	Equivocal	0	Equivocal	0
		Positive	0	Positive	0	Positive	0
		Not evaluable	1	Not evaluable	0	Not evaluable	0
2+	59	Negative	55	Negative	56	Negative	54
		Equivocal	0	Equivocal	0	Equivocal	0
		Positive	1	Positive	3	Positive	3
		Not evaluable	3	Not evaluable	0	Not evaluable	2
3+	27	Negative	2	Negative	0	Negative	0
		Equivocal	0	Equivocal	0	Equivocal	0
		Positive	25	Positive	27	Positive	27
		Not evaluable	0	Not evaluable	0	Not evaluable	0

ASS, Automated slide stainer.

**Table 3:** Characteristics of patients categorized as HER-2 (2+) or 3(+).

Number of patients		148	Hormone receptor status		
Female sex, n (%)		147 (99.3%)			
			ER	0	37
Age	Median	60.3±13.3		1+	10
	Range	33–92		2+	7
				3+	109
Number of specimens	163	PR	0	61	
Right		77		1+	22
Left		86		2+	13
				3+	67
Tumor size		HER-2 IHC	0	36	
	T0-1	89		1+	41
	T2	49		2+	59
	T3-4	25		3+	27
Lymph node					
	Negative	119			
	Positive	44			
