# Supplementary material for: Novel method for rapid in-situ hybridization of HER2 using non-contact alternating-current electric-field mixing
Source: Sci Rep. 2016 Jul 22;6:30034. doi: 10.1038/srep30034 (PMC4957099; doi:10.1038/srep30034)
Supplement: Supplementary Figure 1 [file srep30034-s1.doc]

**Novel method for rapid in-situ hybridization of HER2 using non-contact alternating-current electric-field mixing**

Yoshitaro Saito^1^, Kazuhiro Imai^1^, Ryuta Nakamura^2^, Hiroshi Nanjo^3^, Kaori Terata^1^, Hayato Konno^1^, Yoichi Akagami^2^, and Yoshihiro Minamiya^1^

^1^ Department of Thoracic Surgery, Akita University Graduate School of Medicine

^2^ Akita Industrial Technology Center, Akita, Japan

^3^ Division of Clinical Pathology, Akita University Graduate School of Medicine

**Correspondence:** Kazuhiro Imai, MD PhD

Department of Thoracic Surgery, Akita University Graduate School of Medicine,

1-1-1 Hondo, Akita, 010-8543, Japan

Phone +81 18 884 6132, Fax +81 18 836 2615

Mail: [i-karo@mui.biglobe.ne.jp](mailto:i-karo@mui.biglobe.ne.jp)

**Supplementary Figure 1**

*Detection of Breast Cancer Human Epidermal Growth Factor Receptor 2 (HER2) using rapid Dual-color in Situ Hybridization (RISH)*

**Supplementary Video 1**

*Video showing transformation of the microdroplet’s shape* *as the voltage is switched on and off*
